# Efficacy and safety of PD-1 blockade plus long-course chemoradiotherapy in locally advanced rectal cancer (NECTAR): a multi-center phase 2 study

**DOI:** 10.1038/s41392-024-01762-y

**Published:** 2024-03-11

**Authors:** Zhengyang Yang, Jiale Gao, Jianyong Zheng, Jiagang Han, Ang Li, Gang Liu, Yi Sun, Jie Zhang, Guangyong Chen, Rui Xu, Xiao Zhang, Yishan Liu, Zhigang Bai, Wei Deng, Wei He, Hongwei Yao, Zhongtao Zhang

**Affiliations:** 1 Department of General Surgery, Beijing Friendship Hospital, Capital Medical University, State Key Lab of Digestive Health, National Clinical Research Center for Digestive Diseases, Beijing, China; 2 Department of Gastrointestinal Surgery, The First Affiliated Hospital of Air Force Medical University, Xi’an, China; 3 Department of General Surgery, Beijing Chaoyang Hospital, Capital Medical University, Beijing, China; 4 Department of General Surgery, Beijing Xuanwu Hospital, Capital Medical University, Beijing, China; 5 Department of General Surgery, Tianjin Medical University General Hospital, Tianjin, China; 6 Department of Anorectal, Tianjin People’s Hospital, Tianjin, China; 7 Department of Radiology, Beijing Friendship Hospital, Capital Medical University, Beijing, China; 8 Department of Pathology, Beijing Friendship Hospital, Capital Medical University, Beijing, China; 9 Department of Thoracic Surgery / Institute of Thoracic Oncology, West China Hospital, Sichuan University, Chengdu, China

**Keywords:** Gastrointestinal cancer, Gastrointestinal cancer, Translational research, Outcomes research, Clinical trials

## Abstract

Adding PD-1 blockade in the neoadjuvant regimens for locally advanced rectal cancer (LARC) patients with microsatellite stable (MSS) / mismatch repair-proficient (pMMR) tumors is an attractive, but debatable strategy. This phase 2, multicenter, prospective, single-arm study enrolled patients from 6 centers from June 2021 to November 2022. Locally advanced rectal cancer (LARC, cT_3-4a_N_0_M_0_ and cT_1-4a_N_1-2_M_0_) patients aged ≥18 years with the distance from distal border of tumor to anal verge ≤10 cm (identified by Magnetic Resonance Imaging) were qualified for inclusion. The patients received long-course radiotherapy (50 Gy/25 fractions, 2 Gy/fraction, 5 days/week) and three 21-day cycles capecitabine (850–1000 mg/m2, bid, po, day1–14) and three 21-day cycles tislelizumab (200 mg, iv.gtt, day8) as neoadjuvant. Total mesorectal excision (TME) was 6–12 weeks after the end of radiotherapy to achieve radical resection. A total of 50 patients were enrolled in this study. The pathological complete response rate was 40.0% [20/50, 95% confidence interval (CI): 27.61–53.82%], while 15 (30.0%, 95% CI: 19.1–43.75%), 9 (18.0%, 95% CI: 9.77–30.8%), 2 (4.0%, 95% CI: 1.10–13.46%) patients respectively achieved grade 1, 2, and 3 tumor regression. Treatment-related adverse events (TRAEs) occurred in 28 (56.0%) LARC patients, including 26(52.0%) with grade I-II and 2 (4.0%) with grade III (1 with grade 3 immune-related colitis and 1 with grade 3 rash). PD-1 blockade plus long-course chemoradiotherapy (CRT) showed promising therapeutic effects according to pathological complete response rate and is well-tolerated in LARC patients. A larger randomized controlled study is desired to further validate the above findings.

## Introduction

Rectal cancer is one of the most frequent gastrointestinal malignancies with 253,000 new cases occurred annually in China, accounting for more than 18% worldwide.^1^ For locally advanced rectal cancer (LARC), which is defined here as T3–4 or N positive (T_3-4a_N_0_M_0_ and T_1-4a_N_1-2_M_0_), neoadjuvant chemoradiotherapy (CRT) followed by total mesorectal excision (TME) was preferred as standard treatment by National Comprehensive Cancer Network (NCCN) guidelines to reduce recurrence rate.^2,3^ However, only 11–15% of patients could reach pathological complete response (pCR) after neoadjuvant CRT.^4,5^ LARC patients might benefit from higher pCR rate with fewer complications, higher quality of life, organ preservation, and better oncological prognosis. Consequently, more effective neoadjuvant options against LARC are in the urgent need.

Immune checkpoint inhibitors (ICIs), including programmed cell death ligand 1 (PD-L1), programmed cell death 1 (PD-1), and CTL-associated protein 4 (CTLA-4) blockade, have been proven effective in various solid tumors.^6,7^ ICIs were proven with excellent clinical benefits in locally advanced colorectal cancers (CRCs) of microsatellite instability-high (MSI-H) or mismatch repair-deficient (dMMR) type, which could achieve 75%-100% complete response after neoadjuvant immunotherapy.^8,9^ Unfortunately, MSI-H/dMMR prevalence has been reported with a gradual decrease in its distribution from the proximal colon to the rectum and even less than 5% in LARC.^10,11^ Unsatisfactory efficacies of ICIs were reported in the subtype of microsatellite stable (MSS) / mismatch repair-proficient (pMMR) CRCs.^12,13^ Thus, various improved and collaborative therapeutic trials were carried out to solve this problem of immunotherapies in recent years.^14–16^

Meanwhile, various clinical studies reported that combinations of ICIs with radiotherapy have superior antitumor efficacies and higher response rates.^17,18^ Radiotherapy could reinvigorate exhausted T cells, modulate tumor-associated macrophages (TAMs), inhibit M2 polarization, and reduce tumor burden to affect the therapeutic sensitivity of ICIs like PD-1/PD-L1 inhibitors.^19–21^ As for LARC, the VOLTAGE-A study from Japan reported mild toxicities and a 30% (11/37) pCR rate after treatment with preoperative CRT and the subsequent five cycles of nivolumab in LARC.^22^ Another single-arm trial reported 48.1% (13/27) pCR rate with 96.7% (29/30) treatment-related adverse events (TRAEs) rate using the scheme of short-course radiotherapy (25 Gy/5 f, 5 Gy/f, 5 days), followed by two subsequent 21-day cycles of CAPOX (capecitabine day 1–14 and oxaliplatin day 1) plus camrelizumab (day 1).^23^ The only reported randomized controlled trial in 2021 using FOLFOX (5-fluorouracil, leucovorin, and oxaliplatin) followed by CRT plus pembrolizumab yielded negative results.^12^ The primary endpoint of mean NAR score was 11.53 vs 14.08 (p = 0.26) in the pembrolizumab arm compared to the control arm, while pCR rate was 31.9% (22/69) vs 29.4% (20/68).

However, previous studies did not focus on ICIs plus concomitant and sequential CRT as the neoadjuvant scheme. Additionally, both subtypes of MSI-H/dMMR and MSS/pMMR LARC patients were involved. Taking the consideration above, we reported results from this NECTAR study to identify the safety and efficacy of PD-1 blockades (tislelizumab) plus CRT as neoadjuvant in patients with LARC.^24^

## Results

### Patient characteristics

**Fig. 1:**
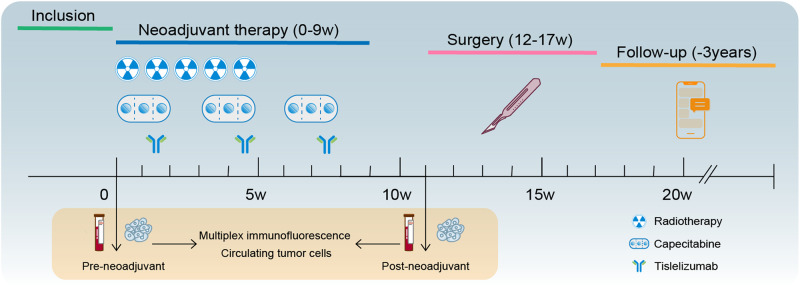
Study design. Patients with cT_3-4_N_0_M_0_ and cT_1-4_N_1-2_M_0_ received long-course radiotherapy (50 Gy/25 f, 2 Gy/f, 5 days/week) and three 21-day cycles capecitabine (850–1000 mg/m2, bid, po, day1–14) plus three 21-day cycles tislelizumab (200 mg, iv.gtt, day8), followed by surgery 6–8 weeks after the end of radiotherapy. The primary efficacy endpoint was the pathological complete response (pCR) rate. Blood and tumor samples were collected before and after neoadjuvant for multiplex immunofluorescence and circulating tumor cells analysis

From June 2021 to November 2022, 60 patients were screened, and 50 patients were enrolled. Detailed schedules and timelines of treatments were shown in Fig. 1. These patients were from 6 third-class hospitals in China of Beijing, Tianjin, and Xi’an. The median age was 62 (26 to 79) years, and the median distance from distal border of the tumor to anal verge was 5.4 (0.6–9.7) cm. Meanwhile, 4 (8.0%), 36 (72.0%), and 10 (20.0%) of these patients were T2, T3, T4 stage, while 18 (36.0%), 19 (38.0%), and 13 (26.0%) were N0, N1, N2 stage correspondingly. The detailed characteristics of these 46 patients are shown in Table 1.

**Table 1:** Baseline Characteristics in Patients

	Patients enrolled (*n* = 50)	Patients received radical resection (*n* = 46)
MSS/pMMR	49 (98%)	46 (100%)
Age [median (range)] year	60 (26–79)	62 (32–79)
Sex		
Male	32 (64%)	29 (63.0%)
Female	18 (36%)	17 (37.0%)
BMI [median (range)]	23.7 (18.8–29.0)	23.7 (18.8–29.0)
ECOG performance status		
0	33 (66%)	30 (65.2%)
1	17(34%)	16 (34.8%)
Distance from distal border of tumor to anal verge [median (range)] cm	5.4 (0.6–9.7)	5.7 (0.6–9.7)
T category (T2/T3/T4)		
T2	4 (8%)	4 (8.7%)
T3	36 (72%)	33 (71.7%)
T4	10 (20%)	9 (19.6%)
N category		
N0	18 (36%)	16 (34.8%)
N1	19 (38%)	19 (41.3%)
N2	13 (26%)	11 (23.9%)
MRF positive	40 (80%)	37 (80.4%)
EMVI positive	12 (24%)	10 (21.7%)
Length of tumor lesion [median (range)] cm	3.70 (1.3–10.4)	3.65 (1.3–10.4)

*MSS* microsatellite stable, *pMMR* mismatch repair-proficient, *BMI* body mass index, *ECOG* Eastern Cooperative Oncology Group, *MRF* mesorectal fascia, *EMVI* extramural venous invasion

### Efficacy and safety

**Fig. 2:**
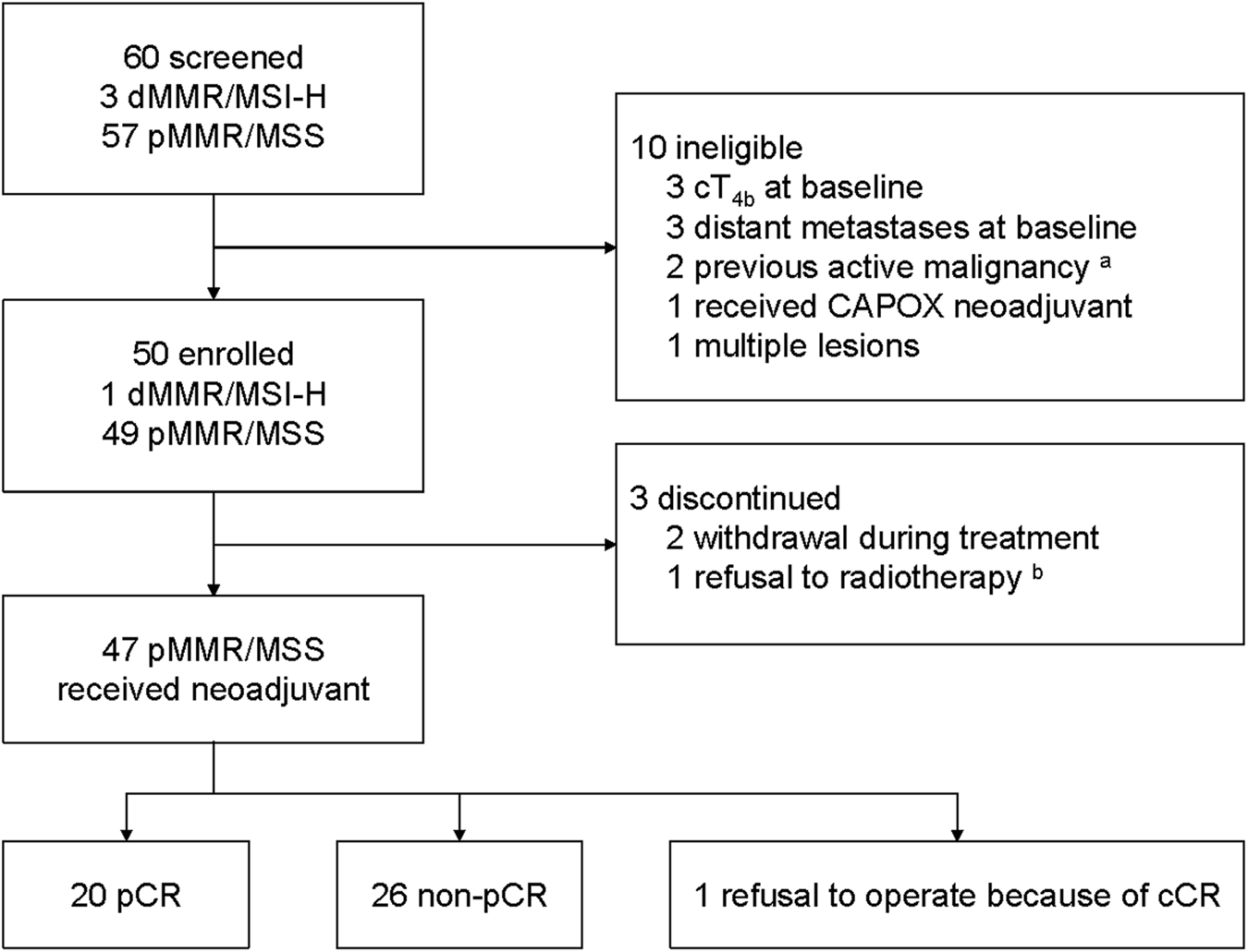
Trial profile. ^a^ One MSI-H patient with ureteral malignancy history and another MSI-H patient with transverse colon cancer history were ineligible. ^b^ One MSI-H patient refused radiotherapy because of her fertility demand at the baseline tumor assessment

**Fig. 3:**
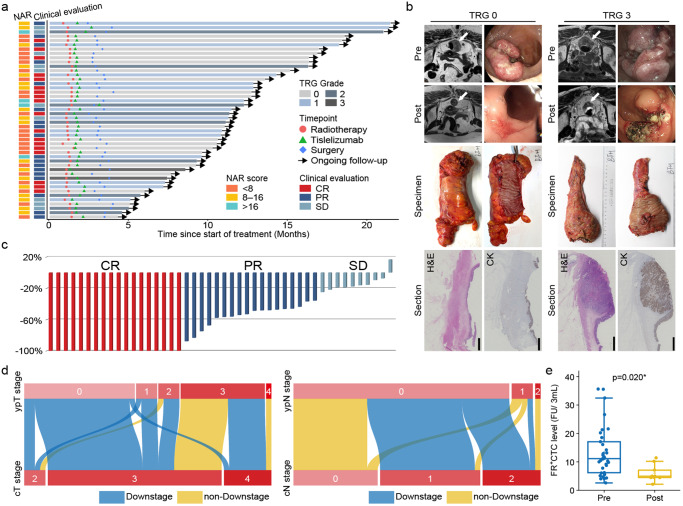
Clinical efficacy. **a** Swimmer plot of 46 patients who reached the primary endpoint in this trial. **b** Representative radiographic image, endoscopic, specimen, and pathological image in patients of TRG 0 and 3. The white arrows represent the lesion sites. **c** Waterfall plot of maximum percent change in tumor size from baseline as measured according to RECIST v1.1. **d** Changes of pre-treatment cT and cN stage to post-treatment ypT and ypN stage in 46 patients who reached the primary endpoint. **e** Dynamic alteration of folate receptor-positive circulating tumor cells (FR^+^ CTCs) levels at pre-neoadjuvant (Pre) and before surgery (Post). The median and interquartile range are shown for each group

A total of 50 patients were enrolled in this study and 47 patients completed the entire course of neoadjuvant (CRT plus 3 cycles tislelizumab). Among them, one patient achieved clinical complete response according to preoperative evaluation and refused the surgical operation. The remaining 46 patients were undergone further radical surgery to evaluate the primary endpoint, pCR (Fig. 2). All these 46 patients were undergone radical surgery with an R0 resection rate of 100%, sphincter-saving resection rate of 89.1% (41/46), and defunctioning ileostomy rate of 31.7% (13/46). The median time from the end of radiotherapy to surgery was 61 (43 to 84) days (Table 2). According to the swimmer plot, the pCR rate was 40.0% [20/50, 95% confidence interval (CI): 27.61–53.82%], while 15 (30.0%, 95% CI: 19.1–43.75%), 9(18.0%, 95% CI: 9.77–30.8%, and 2 (4.0%, 95% CI: 1.10–13.46%) patients reached tumor regression grade 1, 2, and 3 according to AJCC standard.^25^ Interestingly, 21 patients reached ypT0 and 20 of them reached pCR because 1 patient was reported as ypT0N1 through the postoperative pathological evaluation. In addition, 24 (52.2%) patients got low NAR score. In comparison, 18 (39.1%) reached intermediate and 4 (8.7%) reached high (Fig. 3a). Representative response of tumors according to MRI image, endoscopic, specimen, and pathological image in patients of each TRG grade were shown (Fig. 3b and Supplementary Fig. S1). To ensure the accuracy of the primary endpoint, every TRG 0 patient was further evaluated through cytokeratins (CK) staining. As for clinical efficacy assessed according to the Response Evaluation Criteria in Solid Tumors (RECIST) v1.1, 18 (39.1%) complete response, 18 (39.1%) partial response, and 10 (21.7%) stable disease were observed with no progressive disease (Fig. 3c). However, only 11 patients reached the uniformity complete response evaluated by pathological and clinical standards (Supplementary Fig. S2). Meanwhile, 35 (76.1%) patients achieved a downstage of the clinical T category, while 28 (60.9%) of the clinical N category after the CRT plus tislelizumab (Fig. 3d). Additionally, the folate receptor-positive circulating tumor cells (FR^+^ CTCs) levels significantly decreased after the treatment (Fig. 3e). The quality of the excised specimen is next evaluated. According to the mesorectum integrity, 26 (56.5%) postoperative specimens could be evaluated as complete. Circumferential resection margins of all specimens were negative, and 95.7% (44/46) of them with vessel invasion (Supplementary Table S1). As of February 2023, no patients experienced recurrence after a median follow-up of 35.5 weeks (3.7 to 87.7).

**Table 2:** Assessment of efficacy in patients received radical resection

Time from the end of radiotherapy to surgery [median (range)] days	61 (43–84)
R0 resection (yes/no)	46 (100.0%) / 0 (0.0%)
Anal-preserving (yes/no)	41 (89.1%) / 5 (10.9%)
Defunctioning ileostomy	13 (28.3%)
Colostomy	5 (10.9%)
No stomas	28 (60.9%)
AJCC tumor regression grade (0/1/2/3)	20 (43.5%) / 15 (32.6%) / 9 (19.6%) / 2 (4.3%)
ypT category (ypT0/ypT1/ypT2/ypT3/ypT4)	21 (45.7%) / 4 (8.7%) / 4 (8.7%) / 16 (34.8%) / 1 (2.2%)
ypN category (ypN0/ypN1/ypN2)	41 (89.1%) / 4 (8.7%) / 1 (2.2%)
MRI tumor regression grade (0/1/2/3)	18 (39.1%) / 22 (47.8%) / 5 (10.9) / 1 (2.2%)
ycT category (ycT0/ycT1/ycT2/ycT3/ycT4)	18 (39.1%) / 5 (10.9) / 7 (15.2%) / 14 (30.4%) / 2 (4.3%)
ycN category (ycN0/ycN1/ycN2)	40 (87.0%) / 4 (8.7%) / 2 (4.3%)
Clinical evaluation through RECIST	
CR	18 (39.1%)
PR	18 (39.1%)
SD	10 (21.7%)
PD	0 (0%)
ORR	35 (76.1%)
Neoadjuvant rectal score [median (range)]	14.98 (0.94–70.34)

*AJCC* American Joint Committee on Cancer, *MRI* magnetic resonance imaging, *RECIST* Response Evaluation Criteria in Solid Tumors, *CR* complete response, *PR* partial response, *SD* stable disease, *PD* progressive disease, *ORR* objective response rate

The adverse events (AEs) and postoperative complications were all assessed by CTCAE version 4.0 (Supplementary Table S2).^26^ The TRAEs occurred in 28 (56.0%) patients, while the most common TRAEs were fatigue 18 (36.0%), leukopenia 16 (32.0%), and radiation proctitis 14 (28.0%) during the treatment. Among them, 2 (4.0%) cases of ≥ Grade III TRAES were observed with 1 grade 3 immune-related colitis and 1 grade 3 rash. As for postoperative complications, a total of 7 (15.2%) cases of all grades were observed. 2 (4.3%) cases of Grade III rectovaginal fistula and 1 (2.2%) case of intestinal obstruction occurred (Supplementary Table S3). No grade 4/5 adverse event and treatment-related deaths were recorded.

### Altered immune microenvironment after PD-1 blockade plus CRT

**Fig. 4:**
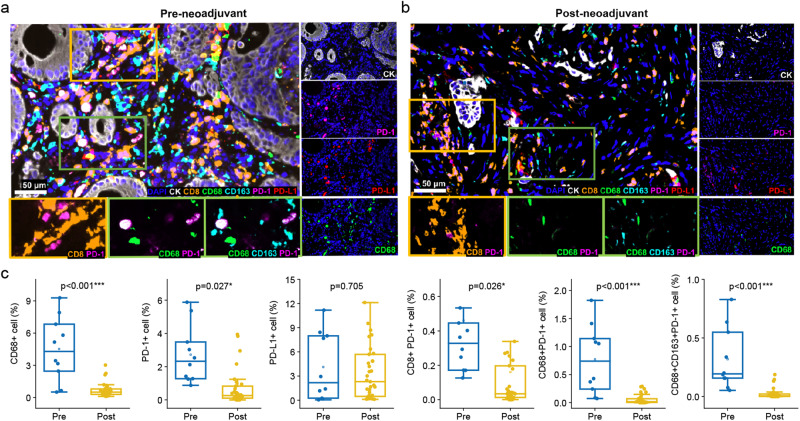
Changes of immune microenvironment after neoadjuvant. **a** Multiplex immunofluorescence staining of the patient before neoadjuvant. **b** Multiplex immunofluorescence staining of the patient after neoadjuvant. **c** Changes of different biomarker-positive cells rate. The median and interquartile range are shown for each group

To further evaluate the changes in the immune microenvironment corresponding to neoadjuvant, multiplex immunofluorescence staining proceeded (Fig. 4a). The PD-1+ cells significantly reduced. In contrast, no significant differences in PD-L1+ cells after treatment with the PD-1 blockade (Fig. 4b). Additionally, CD68+ cells which represented tumor-associated macrophages (TAMs) significantly reduced after PD-1 blockade plus CRT (Fig. 4c). Additionally, significant decrease of T cell exhaustion (CD8 + PD-1 + ), PD-1 positive TAMs (CD68 + PD-1 + ), and PD-1 positive M2 (CD68 + CD163 + PD-1 + ) could also be observed, which might be related to superior therapeutic effect (Supplementary Table S4). Therefore, the infiltration rate of T cell exhaustion, TAMs, PD-1 positive TAMs, and PD-1 positive M2 reduced in the immune microenvironment might be key factors for achieving excellent antitumor efficacy in MSS/pMMR LARC patients after such PD-1 blockade plus CRT therapy.

### Clinical risk factors of response in baseline

To further explore the predictive factors of response in baseline, the baseline lab tests were first evaluated (Supplementary Table S5). However, no significant differences were found between pCR and non-pCR groups. The clinical features were then examined to analyze the risk factors of pCR in baseline (Supplementary Table S6). The univariate analysis suggested that age <50 years and distance from tumor distal border to anal verge <5 cm were related to higher pCR rate. The multivariate analysis, indicated that age <50 years and no elevation of pretreatment CEA were 2 independent influencing factors of pCR, with the the *p* value < 0.1 (age, distance from tumor distal border to anal, and CEA level) in univariate analysis. No significant differences, including distance from tumor distal border to anal, were found in other clinical factors.

Interestingly, the pCR rate in age <50 years as well as CEA level <5 ng/ml reached 100% (3/3) after evaluating 37 patients with complete data of CEA level (Table 3). On the contrary, patients with age ≥50 years and CEA level ≥5 ng/ml could only reach a 16.7% (2/12) pCR rate. Totally, the pCR rate was 85.7% (6/7) in young-onset rectal cancer patients (age<50) while 35.9% (14/39) in others (*p* = 0.014). Additionally, the pCR rate was only 26.7% (4/15) in patients with elevated CEA while 59.1% (13/22) in patients CEA level <5 ng/ml (*p* = 0.046). Consequently, the above results suggested that both age and CEA level might be suffient predictive factors for pCR rate in MSS/pMMR LARC patients after such PD-1 blockade plus CRT therapy.

**Table 3:** Correlation between the pCR rate and both age and CEA level

	CEA level <5 ng/ml	CEA level ≥5 ng/ml	Total
Age < 50 years	100% (3/3)	66.7% (2/3)	83.3% (5/6)
Age ≥50 years	52.6% (10/19)	16.7% (2/12)	38.7% (12/31)
Total	54.5% (13/22)	26.7% (4/15)	

*pCR* pathologic complete response, *CEA* carcinoembryonic antigen

## Discussion

Neoadjuvant CRT followed by radical surgery, the current recognized treatment recommended by National Comprehensive Cancer Network (NCCN), may achieve a limited response, which cannot be completely satisfied in clinic. While ICIs were indicated with promising potency in MSI-H/dMMR solid tumors, the subtype of MSS/pMMR was little benefitted in CRC, especially LARC. As previously reported, the synergistic effect of radiotherapy and ICIs possessed broad prospects, which might be due to a more immunologically active microenvironment.^27^ To investigate the efficacy and safety of combined ICIs and CRT in LARC patients of MSS/pMMR, this prospective, multi-center, phase 2 study was performed. The short-term and interim results were showed in 2022 American Society of Clinical Oncology Annual Meeting (e15599) and European Society of Medical Oncology Asia Congress 2022 (47 P). To our knowledge, this NECTAR study is the first reported multi-center study to investigate PD-1 blocked plus CRT as neoadjuvant therapy against MSS/pMMR LARC patients. Although this is a single-arm, non-randomized study, the primary endpoint of pCR rates reached 40.0%, much higher than the 10%-20% of traditional neoadjuvant therapies.^28,29^ Considering clinical evaluation through RECIST, a CR rate of 39.1% and ORR rate of 76.1 with no PD were also encouraging. The levels of FR^+^ CTCs, correlated with the prognosis of tumor patients, significantly decreased after treatment. In addition, the combination was well-tolerated without unexpected or new safety events.

The ICIs were recommended as the first-line treatment option for patients with MSI-H/dMMR metastatic CRC after the final analysis of the KEYNOTE-177 study reported, whether it is eligible for intensive therapy in NCCN Guidelines Version 2. 2021.^30,31^ Differently, MSS/pMMR could not respond well to ICIs, which might be attributed to severe T cell exhaustion, high density of TAMs, and a higher rate of M2 polarization.^32–34^ For this problem, it has been widely demonstrated that radiotherapy combined with immunotherapy could achieve a synergic effect in the clinic. Radiotherapy could effectively activate the tumor immune microenvironment by inducing tumor antigen release, enhancing tumor cell immunogenicity, activating immune cells, secreting immune factors, and promoting tumor-related antigen presentation.^35^ Such phenomenon might be explained in the literature as turning inherently cold (MSS/pMMR) into hot tumors (MSI-H/dMMR), and thus benefit from ICIs therapies.^27,28^ In this study, T cell exhaustion, density of TAMs, and rate of M2 polarization, which indicate the immuno-suppressive microenvironment, were significantly reduced after the PD-1 blockade plus CRT. Various studies reported that the reinvigoration potential of T cell exhaustion via PD-1 inhibition implicates stronger anti-tumor immunity and better prognosis.^29,36^ PD-1 positive TAMs, which declined after such neoadjuvant, were also recently reported for inhibiting macrophages in the tumor microenvironment.^37^ Given to above results, we hypothesized that CRT might reverse the inhibitory immune microenvironment in MSS/pMMR patients, resulting in the encouraging therapeutic effects.

Besides, a few studies mentioned the combination regimens of CRT and ICIs as neoadjuvant treating MSS/pMMR LARC. In VOLTAGE-A study, nivolumab (5 cycles) followed by CRT reached a 30% pCR rate in MSS/pMMR LARC patients.^22^ Chemotherapy plus concurrent radiotherapy was suggested more effective than sequential radiotherapy in non–small cell lung cancer, but was not reported in CRCs.^38,39^ Additionally, the best tumor response occurs at 8 weeks after completion of a long-course radiotherapy according to NCCN Guidelines of Rectal Cancer, Version 2.2022.^40^ Given these results, concomitant and sequential CRT was chosen in this study instead of a sequential plan. As expected, this study acquired a higher pCR rate than the VOLTAGE-A study (40.0% vs 29.7%). In NRG-GI002 study, patients in the experimental arm received 6 cycles of FOLFOX followed by CRT in combination with 200 mg of pembrolizumab with grade 3 to 4 adverse events of 48.2% (39/81). Another phase 2 single-center trial from China reported patients received short-course radiotherapy (25 Gy/5 f, 5 Gy/f, 5 days) followed by two subsequent 21-day cycles of CAPOX (capecitabine day 1–14 and oxaliplatin day 1) plus camrelizumab (day 1) reached pCR rate of 46.2% (12/26). However, the overall TRAEs occurred 96.7% (29/30) with 8 (10%) grade 3.^23^ Comparatively, the rate in this study was 56.0% (28/50) and 4.0% (2/50). Considering the addition of ICIs, single capecitabine without intravenous chemotherapy (oxaliplatin) might be better tolerated than CAPOX in these LARC patients. Based on the above reasoning, the long-course CRT plus PD-1 blockade was chosen as the neoadjuvant therapy.

Preliminary study has shown that low-dose chemotherapy is usually more effective in immune stimulation than more intense chemotherapies.^41^ It is reported that chemotherapy could selectively killed antitumor immune cells (CXCL13 + T cells) and thus further weaken the response of ICIs.^41^ CD8 + T cells, as the most important immune cells ICIs produce the enhancing anti-tumor immunity, needed to fully recover to initial levels within 1 year after chemotherapy.^42^ In addition, preliminary findings from an ongoing clinical study suggest that chemotherapy after ICIs is more efficacious than ICIs after chemotherapy in patients with BRAF-wildtype metastatic melanoma.^43^ As the only reported randomized controlled trials, the NRG-GI002 study yielded negative results both in pCR rate (31.9% vs 29.4%) and NAR score (11.53 vs. 14.08) between the pembrolizumab arm and the control arm12. As a comparison, this NECTAR study obtained higher response rates. Thus, we speculate that this phenomenon may be due to the killing impact of 6 cycles FOLFOX on immune cells like CD8 + T cell. Low-dose chemotherapy may have a positive promoting effect on immunotherapy, while large-dose may lead to the inactivation of the immune cells, thereby limiting the effectiveness of immunotherapy. Therefore, although there are evidences to confirm the effectiveness of chemotherapy plus immunotherapy, further clinical studies with higher levels of evidence are still needed for the selection of chemotherapy timing and dosage.

As the primary endpoint of this study, pCR was defined as no residual tumor cells on the histologic examination of surgical specimens according to AJCC 8th edition. Reaching pCR is considered to be associating with a low probability of local recurrence and distant failure, which indicates superior long-term survival.^44^ As reported in a classical pooled analysis, patients with pCR reached a 5-year local recurrence of 2.8% compared with non-pCR (9.7%). Similarly, more competitive 5-year disease-free survival (83.3% vs 65.6%) and overall survival (87.6% vs 76.4%) were observed in the pCR group than in patients without pCR.^45^ The CAO/ARO/AIO-94 trial reported that 10-year cumulative incidence of distant metastasis (10.5%, 29.3%, and 39.6% in complete, intermediate, and poor regression) and disease-free survival (89.5%, 73.6%, and 63% in complete, intermediate, and poor regression) were significantly associated with tumor regression.^46^ Therefore, pCR rate was chosen as the primary endpoint in this study to evaluate the efficacy of neoadjuvant more accurately.

When LARC patients achieve clinical complete response (cCR), the watch-and-wait strategy and local excision could be optional to preserve organs and improve quality of life.^47^ However, owing to no objective evaluation criteria, the reliability of concordance between pCR and cCR was still questioned.^48,49^ Although pCR is considered the more accurate prognostic indicator, it cannot be identified preoperation because it is determined through surgical pathology only. This study also found that 38.9% (7/18) of patients who reached cCR preoperation had residual cancer after resection, while another patient was excluded because of refusing surgery after cCR. Thus, screening the group of beneficiaries from this PD-1 blockade plus CRT neoadjuvant is a critical question of this study. Previous studies reported that higher CD8/eTreg ratio, PD-L1 combined positive score (CPS), tumor mutational burden (TMB), and CD8 + PD-1 + T cell infiltration predicted better response in pMMR tumors.^13,22,23^ This study discovered marvelously that patients aged <50 years and CEA level <5 ng/ml were independent predictive factors to pCR, while patients reached 100% pCR rate after meeting both conditions simultaneously. Several previous studies reported that baseline CEA level was inversely correlated with pCR rate after CRT, but no report investigated whether CEA level could predict the response to ICIs.^50,51^ As the most frequently accepted CRC marker, we expect baseline CEA level as a reliable predictor for therapeutic effects of PD-1 blockade plus CRT. For patients aged <50 years, some pathological characteristics, such as low tumor-infiltrating lymphocytes and poor tumor differentiation were reported.^52^ These conditions might be reversed by CRT, and further sensitized the treatment effect of immunotherapy.

In conclusion, PD-1 blockade plus CRT showed an encouraging curative effect according to the pCR rate with mild toxicities in LARC patients although the study was conducted with a relatively small sample size and the long-term survival outcomes need to be revealed in the follow-ups. To further support the findings of PD-1 blockade plus CRT with higher-level pieces of evidence, a phase III, multi-center, open-label, three-arm, randomized controlled trial (NCT05245474) is undergoing.^53^ We expect that patients aged <50 years and (or) with CEA level <5 ng/ml are more likely to benefit from organ preservation through a watch-and-wait strategy or local excision in the future.

## Materials and Methods

### Ethics approval and consent to participate

This study collected serial blood and tissue sample from the human subjects. The first-edition protocol^24^ and amendment^54^ have been reported previously, which were approved by the Ethics Committee of Beijing Friendship Hospital, Capital Medical University on Mar 30, 2021, and Feb 25, 2022, respectively. This study was carried out in accordance with the principles of the Helsinki Declaration. All patients provided written, informed consent before inclusion.

### Study design and participants

This trial (ClinicalTrials.gov NCT04911517) was a prospective, multicenter, open-label, phase 2, single-arm clinical trial to evaluate the safety and efficacy of NEoadjuvant Chemoradiotherapy plus Tislelizumab followed by TME treating locally Advanced Rectal cancer (NECTAR). Consecutive LARC (cT_3-4a_N_0_M_0_ and cT_1-4a_N_1-2_M_0_) patients aged ≥18 years with the distance from distal border of tumor to anal verge ≤10 cm (identified by MRI) were qualified for inclusion. Mainly exclusion criteria included pregnant or lactating women, present or previous active malignancies (except diagnosis of LARC this time), acquired or congenital immune deficiency and so on. Detailed inclusion and exclusion criteria were shown in Supplementary Table S7. To better align with clinical practice, the protocol amendment was undertaken with the inclusion of mid-to-low LARC (distance from distal border of tumor to anal verge ≤10 cm) and the judgment of tumor regression through American Joint Committee on Cancer (AJCC) standard.

### Procedures

Baseline assessments of complete medical history and physical examination, colonoscopy, MRI (rectal), and CT (chest, abdominal, and pelvic) were necessary in each patient. All enrolled patients received three 21-day cycles PD-1 blockade (tislelizumab 200 mg, iv.gtt, day8) with long-course CRT (50 Gy/25 f, 2 Gy/f, 5 days/week plus three 21-day cycles capecitabine 850–1000 mg/m^2^, bid, po, day1–14) as neoadjuvant. Afterwards, patients had 2 weeks of rest (week 10–11). Clinical efficacy was then evaluated (at least 6 weeks after the end of radiotherapy) with physical examination, colonoscopy, MRI (rectal), and CT (chest, abdominal, and pelvic) pre-operation. These LARC patients then received TME for radical resection 6–12 weeks after the end of radiotherapy (week 12–17). Postoperative adjuvant therapies were nonuniformly specified and decided according to clinical experiences. Postoperative follow-ups were carried out every 3 months the first year and every 6 months the second year until 3 years or to the date of death by clinicians through regular outpatient visits and/or telephone.

The MSI status were detected through polymerase chain reaction with fluorescent primers‐capillary electrophoresis, while MMR status through immunohistochemistry at the time of baseline. Additionally, circulating tumor cells (CTCs) were analyzed and quantified at the time of pre- and postneoadjuvant using the FR^+^CTCs Detection Kit (Geno Biotech Co Ltd) approved by the National Medical Products Administration. The blood samples were analyzed at the time of baseline and pre-operation to collect the information, including total white blood cells (WBCs), neutrophils, lymphocytes, eosinophils, basophils, monocytes, hemoglobin (Hb), platelet (PLT), C-reactive protein (CRP), K, Na, Ca, glucose, carcinoembryonic antigen (CEA), and CA199. The neutrophil-to-lymphocyte ratio (NLR) and platelet-to-lymphocyte ratio (PLR) were respectively calculated as the neutrophils or the patelets divided by lymphocytes.The lymphocyte-to-monocyte ratio (LMR) was calculated as the lymphocytes divided by monocytes. The systemic immune-inflammatory index (SII) was calculated as platelets multiplied by NLR.

### Assessment

To ensure the accuracy of enrolled patients and clinical evaluation, all pre-neoadjuvant and pre-operative MRI images were uploaded to the network data registration. Further independent central reviews were reported and issued by two professional radiologists using standardized radiological reports before and after neoadjuvant (Supplementary Table S8). To strictly demand surgical quality, all participating surgeons were acquired to submit at least 3 unedited surgical video recordings for central review. Two reviewers (Prof Hongwei Yao and Prof Zhongtao Zhang) independently assessed these videos for pre-qualifying. Additionally, these surgeons were also required with experience of at least 30 surgical procedures.

The primary endpoint was pCR rate, defined as the proportion of patients with pCR (ypT0N0). The pathological tumor regression was assessed according to the standards in the 8th Edition of the American Joint Committee on Cancer Guidelines (AJCC). Tumor regression grade (TRG) 0, 1, 2, and 3 indicated no residual tumor cells, single or small groups of cells, residual cancer with a desmoplastic response, and minimal evidence of tumor response, correspondingly. For more accurate assessments, the fresh, intact specimen were collected and evaluated by surgeons and pathologists together to identify the tumor site. The located tumor sites including up to 2 cm above and below them were sliced every 3 mm for pathological evaluation. All tissue sections were processed with hematoxylin and eosin (H&E), and cytokeratins (CK) staining. All these slices of enrolled patients were submitted to two pathologists (Prof Guangyong Chen and Prof Rui Xu) for independent central review to confirm pCR. All lymph nodes were examined, and a minimum of 10 lymph nodes were acquired for adequate assessment of the N stage. As for post-neoadjuvant, less than 10 was also allowed after a repeated and careful search for lymph nodes. Numbers of lymph node metastases as well as total lymph nodes were reported according to the standardized pathological report (Supplementary Table S9).^55^

The neoadjuvant rectal (NAR) score was calculated through pathological (T and N stages) and clinical dimension (T stages), which higher score represents poorer prognosis.^56,57^ High (>16), intermediate (8–16), and low (<8) NAR score were correspondingly defined. The clinical tumor response was assessed pre-operation according to Response Evaluation Criteria in Solid Tumors (RECIST) v1.1 through MRI.^58^ Partial response (PR) rate plus complete response (CR) rate were objective response rate (ORR). R0 resection rate was regarded as percentage of negative margin microscopically.

Adverse events (AEs) and postoperative complications were all performed using the Common Terminology Criteria for Adverse Events (CTCAE) v4.0. Monitoring of AEs were performed every 2 weeks during neoadjuvant. Management of AEs referred to the Society for Immunotherapy of Cancer (SITC) toxicity management working group.^59^

### Multiplex immunofluorescence (mIF) staining

Tumor specimens were acquired from pre-neoadjuvant colonoscopy and surgical resection for mIF staining. All tissues were formalin-fixed, paraffin-embedded, and sectioned as slides of 4-μm thickness. The slides were deparaffinized 30 mins in xylene further rehydrated 5 mins in absolute ethyl alcohol, 5 mins in 95% ethyl alcohol and 2 mins in 75% ethyl alcohol immediately. Then, slides were washed three times and submersed in boiling EDTA buffer (ZSGB Biotech) 15 mins for epitope retrieval. Antibody diluent/blocking (Alpha X Biotech) was used for blocking.

The mIF experiments were performed and analyzed with antibodies against CD8 (ZM0508, ZSGB Biotech), CD68 (ZM0060, ZSGB Biotech), CD163 (ZM0428, ZSGB Biotech), PD-1 (ZM0381, ZSGB Biotech), PD-L1 (13684 S, Cell Signaling Tech), and CK (ZM0069, ZSGB Biotech). All primary antibodies were cultured at 37 °C (60 mins) then slides were cultured at 37 °C (10 mins). The AlphaTSA Multiplex IHC Kits (AXT37025011) were then used for visualization. After each staining cycle, both primary and secondary antibodies were removed through heat-induced epitope retrieval. Nuclei of slides were finally counterstained (5 mins) through DAPI and enfolded in a mounting medium. Multispectral images were scanned with Axioscan 7 Microscopy (Zeiss), while cells of interest were quantified through HALO image analysis (Indica Labs).

### Sample size and statistical analysis

This study is designed as a multicenter, single-arm phase II clinical trial. The pCR rate of single preoperative NCRT is assumed to be about 15% according to previous studies. On the other hand, the expectant pCR rate in this trail will be 40%. The required sample size was calculated to be 50 patients with 80% power and 95% confidence intervals. Moreover, 10% loss of follow-up rate is also considered. Such sample size was calculated using PASS software (version 15).

Statistical analysis was performed using the SPSS software (v24.0, IBM). Unpaired t-test with Welch’s correction was used to analyze continuous variables, which were presented as the mean ± standard deviation. The chi-square test with Fisher’s exact test was used to analyze categorical variables, which were presented as numbers and percentages. All analyses were 2-tailed. The *p* value < 0.05 was considered statistically significant (*p < 0.05, ** *p* < 0.01, and *** *p* < 0.001). The *p* value < 0.1 between the two groups in univariate analysis was assessed in the multivariate analysis to identify independent influencing factors. Further multivariate analysis was performed through logistic regression analysis (stepwise regression).

### Supplementary information

Supplementary Information

## Data Availability

The raw data could be available for scientific purpose by sending requests to the corresponding author Hongwei Yao (yaohongwei@ccmu.edu.cn) within 5 years after this paper’s publication.
