# Supplementary material for: Efficacy and safety of PD-1 blockade plus long-course chemoradiotherapy in locally advanced rectal cancer (NECTAR): a multi-center phase 2 study
Source: Signal Transduct Target Ther. 2024 Mar 11;9:56. doi: 10.1038/s41392-024-01762-y (PMC10925604; doi:10.1038/s41392-024-01762-y)
Supplement: Supplementary file 1 — Supplementary Information [file 41392_2024_1762_MOESM1_ESM.docx]

Supplementary Materials for

Efficacy and safety of PD-1 blockade plus long-course chemoradiotherapy in locally advanced rectal cancer (NECTAR): a multi-center phase 2 study

Zhengyang Yang, Jiale Gao, Jianyong Zheng, Jiagang Han, Ang Li, Gang Liu, Yi Sun, Jie Zhang, Guangyong Chen, Rui Xu, Xiao Zhang, Yishan Liu, Zhigang Bai, Wei Deng, Wei He, Hongwei Yao, Zhongtao Zhang

Correspondence to: Zhongtao Zhang (zhangzht@ccmu.edu.cn), Hongwei Yao (yaohongwei@ccmu.edu.cn), Wei He (hewei@wchscu.cn)

**This PDF file includes:**

Figures. S1 to S2

Tables S1 to S9

Figure. S1. Representative endoscopic, radiographic image, specimen, and pathological image in patients of TRG 1 and 2. The white arrows represent the lesion sites. The scale bar is 2.5 mm.


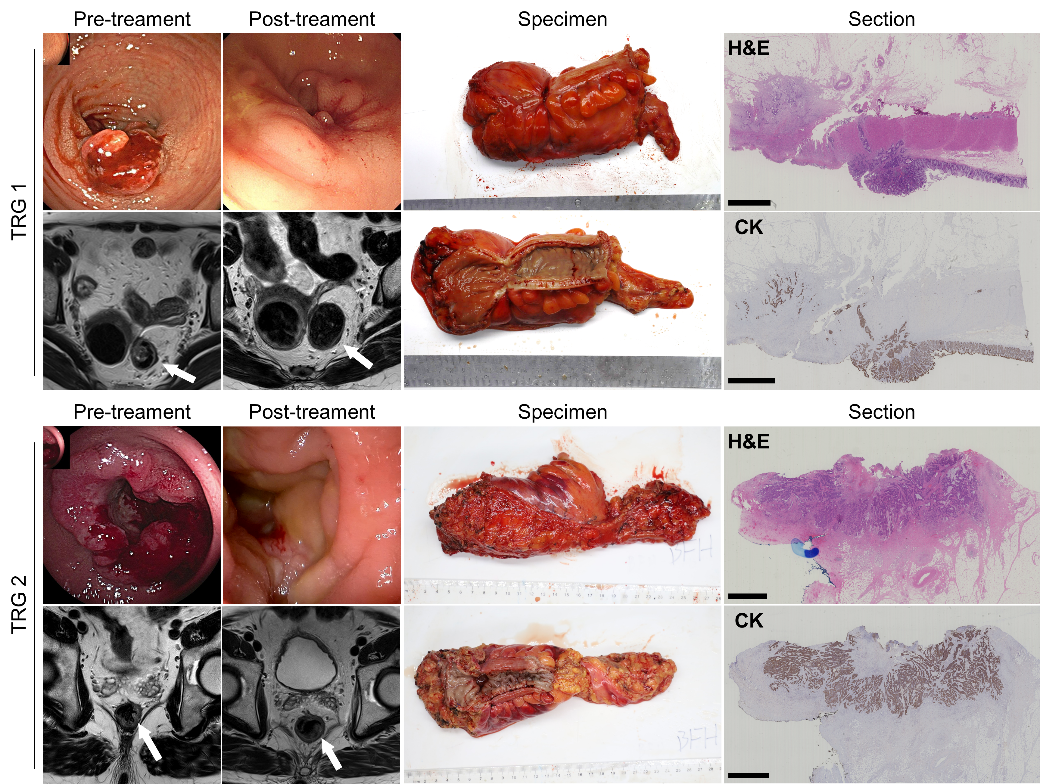


Figure. S2. Overlap of complete response evaluated by pathological and clinical standards.


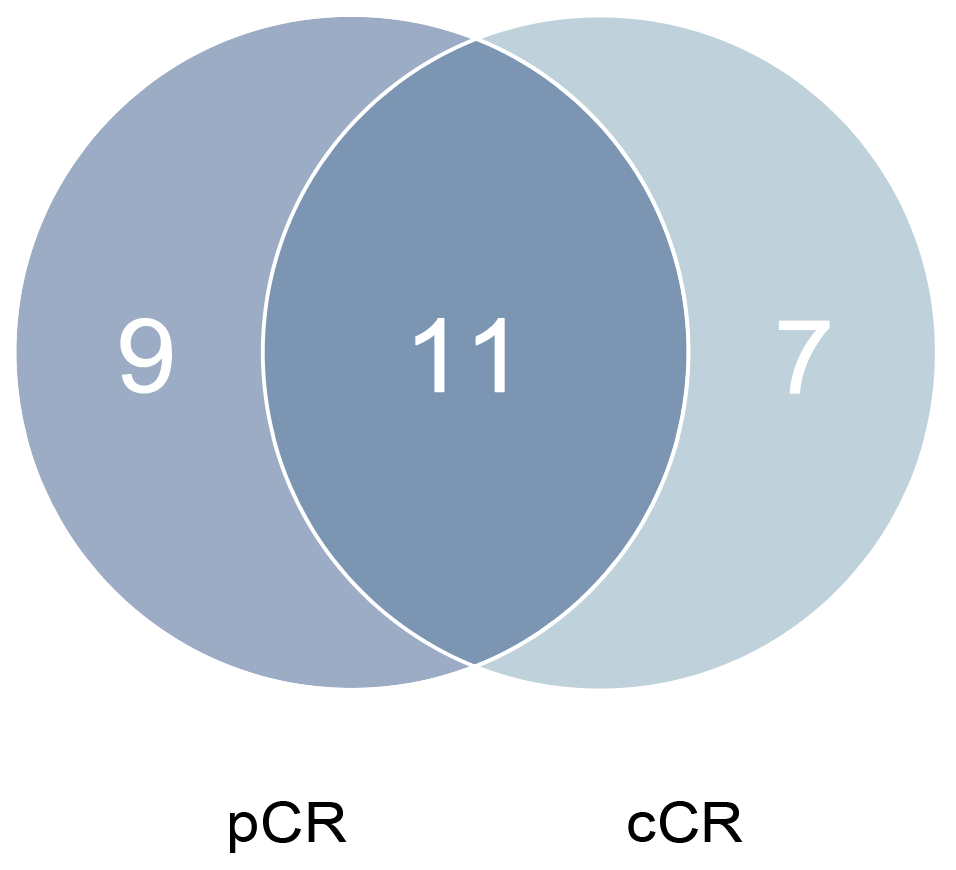


Table S1. Postoperative pathological results in patients who have undergone surgery

| Macroscopic assessment of resection specimen |  |
| --- | --- |
| Complete [n (%)] | 26 (56.5%) |
| Nearly complete [n (%)] | 18 (39.1%) |
| Incomplete [n (%)] | 2 (4.3%) |
| Circumferential resection margin |  |
| Negative [n (%)] | 46 (100%) |
| Positive [n (%)] | 0 (0%) |
| Length of tumor lesion [median (range)] | 1.8 (0.0-5.0) cm |
| Width of tumor lesion [median (range)] | 1.1 (0.0-4.0) cm |
| Number of total lymph nodes [median (range)] | 12 (4, 34) |
| Number of lymph node metastases [median (range)] | 0 (0, 4) |
| Vessel invasion | 44 (95.7%) |
| T category |  |
| ypT0 [n (%)] | 21 (45.7%) |
| ypT1 [n (%)] | 4 (8.7%) |
| ypT2 [n (%)] | 4 (8.7%) |
| ypT3 [n (%)] | 16 (34.8%) |
| ypT4 [n (%)] | 1 (2.2%) |
| N category |  |
| ypN0 [n (%)] | 41 (89.1%) |
| ypN1 [n (%)] | 4 (8.7%) |
| ypN2 [n (%)] | 1 (2.2%) |
| AJCC tumor regression grade |  |
| 0 [n (%)] | 20 (43.5%) |
| 1 [n (%)] | 15 (32.6%) |
| 2 [n (%)] | 9 (19.6%) |
| 3 [n (%)] | 2 (4.3%) |

Table S2. Safety assessed of patients enrolled in this study through the Common Terminology Criteria for Adverse Events (CTCAE) version 4.0.

| Treatment-related adverse events | Grade I-II [n (%)] | ≥Grade III [n (%)] | All grade [n (%)] |
| --- | --- | --- | --- |
| Fatigue | 18(36.0%) | 0(0.0%) | 18(36.0%) |
| Pruritus | 12(24.0%) | 0(0.0%) | 12(24.0%) |
| Radiation proctitis | 14(28.0%) | 0(0.0%) | 14(28.0%) |
| Nausea | 9(18.0%) | 0(0.0%) | 9(18.0%) |
| Leukopenia | 16(32.0%) | 0(0.0%) | 16(32.0%) |
| Rash | 10(20.0%) | 1(2.0%) | 11(22.0%) |
| Diarrhea | 13(26.0%) | 0(0.0%) | 13(26.0%) |
| Anemia | 6(12.0%) | 0(0.0%) | 6(12.0%) |
| Abdominal pain | 7(14.0%) | 0(0.0%) | 7(14.0%) |
| Neutropenia | 8(16.0%) | 0(0.0%) | 8(16.0%) |
| Arthralgia | 3(6.0%) | 0(0.0%) | 3(6.0%) |
| Alanine transaminase increased | 2(4.0%) | 0(0.0%) | 2(4.0%) |
| Immune-related colitis | 0(0.0%) | 1(2.0%) | 1(2.0%) |
| Chest pain | 1(2.0%) | 0(0.0%) | 1(2.0%) |
| Hypothyroidism | 1(2.0%) | 0(0.0%) | 1(2.0%) |
| Hyperthyroidism | 1(2.0%) | 0(0.0%) | 1(2.0%) |
| Skin depigmentation | 1(2.0%) | 0(0.0%) | 1(2.0%) |
| Bullous pemphigoid | 1(2.0%) | 0(0.0%) | 1(2.0%) |
| Lumbalgia | 1(2.0%) | 0(0.0%) | 1(2.0%) |
| Herpes | 1(2.0%) | 0(0.0%) | 1(2.0%) |
| Facial swelling | 1(2.0%) | 0(0.0%) | 1(2.0%) |
| Total | 26(52.0%) | 2(4.0%) | 28(56.0%) |

Table S3. Postoperative complications assessed of patients received radical resection through the Common Terminology Criteria for Adverse Events (CTCAE) version 4.0.

| Postoperative complications | Grade I-II [n (%)] | ≥Grade III [n (%)] | All grade [n (%)] |
| --- | --- | --- | --- |
| Intestinal obstruction | 1 (2.2 %) | 1 (2.2 %) | 2 (4.3 %) |
| Anastomotic leakage | 2 (4.3 %) | 0 (0.0 %) | 2 (4.3 %) |
| Rectovaginal fistula | 0 (0.0 %) | 2 (4.3 %) | 2 (4.3 %) |
| Bleeding | 1 (2.2 %) | 0 (0.0 %) | 1 (2.2 %) |
| Total | 4 (8.7 %) | 3 (6.5 %) | 7 (15.2 %) |

Table S4. Comparison of different markers between baseline and preoperative multiplex immunofluorescence.

|  | Baseline | Post-treatment | p-value |
| --- | --- | --- | --- |
| CD8+ (Mean ± SD) % | 4.9848±2.5161 | 5.4075±3.8035 | 0.743 |
| CD68+ (Mean ± SD) % | 4.5145±2.9798 | 0.6845±0.6251 | <0.001*** |
| CD163+ (Mean ± SD) % | 5.2923±5.3809 | 5.0067±2.6566 | 0.816 |
| PD-1+ (Mean ± SD) % | 2.7221±1.7421 | 0.9912±2.2089 | 0.027* |
| PD-L1+ (Mean ± SD) % | 4.1204±4.2252 | 3.5909±3.7818 | 0.705 |
| CD8+PD-1+ (Mean ± SD) % | 0.4604±0.5063 | 0.1603±0.3167 | 0.026* |
| CD8+PD-L1+ (Mean ± SD) % | 0.7001±0.7715 | 0.5705±0.8646 | 0.671 |
| CD68+PD-1+ (Mean ± SD) % | 0.7712±0.6097 | 0.0550±0.0847 | <0.001*** |
| CD68+PD-1+CD163- (Mean ± SD) % | 0.4534±0.5572 | 0.0331±0.0589 | <0.001*** |
| CD68+PD-1+CD163+ (Mean ± SD) % | 0.3177±0.2636 | 0.0219±0.0440 | <0.001*** |
| CD68+CD163- (Mean ± SD) % | 2.0673±2.2008 | 0.3614±0.3844 | <0.001*** |
| CD68+CD163+ (Mean ± SD) % | 2.4471±1.5707 | 0.3857±0.5302 | <0.001*** |

Table S5. Comparison of pCR and non-pCR patients in baseline lab test.

|  | pCR | non-pCR | p-value |
| --- | --- | --- | --- |
| WBCs (Mean ± SD) 10^9/L | 6.27±1.59 | 6.61±1.56 | 0.500 |
| Neutrophils (Mean ± SD) 10^9/L | 3.99±1.28 | 4.50±1.15 | 0.219 |
| Lymphocytes (Mean ± SD) 10^9/L | 3.06±5.01 | 2.61±3.83 | 0.760 |
| Eosinophils (Mean ± SD) 10^9/L | 0.09±0.06 | 0.14±0.10 | 0.073 |
| Basophils (Mean ± SD) 10^9/L | 0.02±0.04 | 0.02±0.04 | 0.712 |
| Monocytes (Mean ± SD) 10^9/L | 0.83±2.00 | 0.38±0.08 | 0.324 |
| Hb (Mean ± SD) g/L | 132.44±15.25 | 122.80±42.58 | 0.368 |
| PLT (Mean ± SD) 10^9/L | 246.22±73.25 | 254.76±64.38 | 0.700 |
| CRP (Mean ± SD) mg/L | 5.54±12.79 | 2.81±3.60 | 0.390 |
| ALB (Mean ± SD) g/L | 41.09±2.58 | 41.30±3.36 | 0.850 |
| GLB (Mean ± SD) g/L | 30.53±2.90 | 29.94±4.50 | 0.688 |
| K (Mean ± SD) mmol/L | 4.19±0.52 | 4.23±0.32 | 0.797 |
| Na (Mean ± SD) mmol/L | 140.36±2.23 | 140.79±2.04 | 0.587 |
| Ca (Mean ± SD) mg/dL | 2.35±0.10 | 2.32±0.11 | 0.488 |
| Glucose (Mean ± SD) mmol/L | 7.39±4.13 | 5.89±1.48 | 0.185 |

Table S6. Univariate and multivariate analysis of risk factors associated with pCR in baseline.

|  | Univariate analysis | | | Multivariate analysis | | |
| --- | --- | --- | --- | --- | --- | --- |
|  | pCR | non-pCR | p-value | OR | 95% CI | p-value |
| Age [n (%)] |  |  | 0.014* | 20.385 | 1.465 ~ 283.690 | 0.025* |
| ≥50 years | 14(70.00) | 25(96.15) |  |  |  |  |
| < 50 years | 6(30.00) | 1(3.85) |  |  |  |  |
| Sex [n (%)] |  |  | 0.108 |  |  |  |
| Female | 10(50.00) | 7(26.92) |  |  |  |  |
| Male | 10(50.00) | 19(73.08) |  |  |  |  |
| BMI (kg/m2) | 23.20±2.62 | 24.18±2.66 | 0.216 |  |  |  |
| Distance from distal border of tumor to anal verge [n (%)] |  |  | 0.026* | 0.189 | 0.035 ~ 1.025 | 0.053 |
| Mid (5-10 cm) | 9(45.00) | 20(76.92) |  |  |  |  |
| Low (< 5cm) | 11(55.00) | 6(23.08) |  |  |  |  |
| T category n (%) |  |  | 0.092 |  |  |  |
| 2 | 2(10.00) | 2(7.69) |  |  |  |  |
| 3 | 17(85.00) | 16(61.54) |  |  |  |  |
| 4 | 1(5.00) | 8(30.77) |  |  |  |  |
| N category n (%) |  |  | 0.842 |  |  |  |
| 0 | 7(35.00) | 9(34.62) |  |  |  |  |
| 1 | 9(45.00) | 10(38.46) |  |  |  |  |
| 2 | 4(20.00) | 7(26.92) |  |  |  |  |
| MRF n (%) |  |  | 0.494 |  |  |  |
| Negative | 17(85.00) | 20(76.92) |  |  |  |  |
| Positive | 3(15.00) | 6(23.08) |  |  |  |  |
| EMVI n (%) |  |  | 0.638 |  |  |  |
| Negative | 5(25.00) | 5(19.23) |  |  |  |  |
| Positive | 15(75.00) | 21(80.77) |  |  |  |  |
| CEA level n (%) |  |  | 0.052 | 0.156 | 0.025 ~ 0.967 | 0.046* |
| <5 ng/ml | 13(76.47) | 9(45.00) |  |  |  |  |
| ≥5 ng/ml | 4(23.53) | 11(55.00) |  |  |  |  |
| NLR | 2.38±1.19 | 2.68±0.88 | 0.378 |  |  |  |
| PLR | 146.54±67.13 | 152.97±44.72 | 0.730 |  |  |  |
| LMR | 5.20±2.18 | 4.67±1.20 | 0.354 |  |  |  |
| SII (Mean ± SD) | 604.99±357.85 | 701.15±345.03 | 0.412 |  |  |  |

Table S7. Inclusion and exclusion criteria.

| **Inclusion criteria:** |
| --- |
| 1. Patients have been fully aware of the content of this study and signed the informed consent voluntarily; |
| 2. Patients with rectal cancers must satisfied all the following conditions: |
| 1) Stage II/III LARC (cT_3-4a_N_0_M_0_ and cT_1-4a_N_1-2_M_0_); |
| 2) Tumor distal located ≤ 10 cm from anal verge (MRI diagnosed); |
| 3. Patients regardless of gender with aged ≥18 years and ECOG score of 0 or 1; |
| 4. Physical and viscera function of patients can withstand major abdominal surgery; |
| 5. Patients are willing and able to follow the study protocol during the study; |
| 6. Patients give consent to the use of blood and pathological specimens for study; |
| 7. Within 28 days prior to enrolment, we must confirm a negative serological pregnancy test for child-bearing age women and they agree to use effective contraception for the duration of drug use and for 60 days after the last dose. |
| **Exclusion criteria:** |
| 1. Patients have a present or previous active malignancy except the diagnosis of rectal cancer this time; |
| 2. Patients underwent major surgery within 4 weeks prior to study treatment; |
| 3. Patients have any condition affects the absorption of capecitabine through gastrointestinal tract; |
| 4. Patients have severe uncontrolled recurrent infections, or other severe uncontrolled concomitant diseases; |
| 5. Patients who are allergic to any of the ingredients under study; |
| 6. Patients with severe concomitant diseases with estimated survival ≤ 5 years; |
| 7. Patients with present or previous moderate or severe liver and kidney damage presently or previously; |
| 8. Patients have received other study medications or any immunotherapy currently or in the past; |
| 9. Patients preparing for or previously received organ or bone marrow transplant; |
| 10. Patients who received immunosuppressive or systemic hormone therapy for immunosuppressive purposes within 1 month prior to the initiation of study therapy; |
| 11. Patients with congenital or acquired immune deficiency (such as HIV infection); |
| 12. If patients with a history of uncontrolled epilepsy, central nervous system disease or mental disorder, the investigator will determine whether the clinical severity prevents the signing of informed consent or affects the patient's oral medication compliance; |
| 13. Patients with other factors that may affect the study results or cause the study to be terminated midway, such as alcoholism, drug abuse, other serious diseases (including mental illness) requiring combined treatment and severe laboratory examination abnormalities. |
| 14. Pregnant or lactating women |

Table S8. Standardized MRI report.

| Position (Distance to anal verge) | ______cm |
| --- | --- |
| Length of tumor | ______cm |
| T-status | T0: no evidence of primary tumor  Tis: carcinoma in situ: intraepithelial or invasion of lamina propria  T1: tumor invades submucosa  T2: tumor invades muscularis propria  T3a: tumor invades beyond muscularis <1mm  T3b: tumor invades beyond muscularis 1-5mm  T3c: tumor invades beyond muscularis 5-15mm  T3d: tumor invades beyond muscularis <15mm  T4a: tumor invades directly into other organs or structures  T4b: tumor perforates visceral peritoneum |
| N-status | N0: no lymph node or lymph node < 5 mm without malignant criteria  N+:  lymph node < 5mm with all malignant criteria  lymph node 5-9mm with ≥ 2 malignant criteria  lymph node ≥ 9mm (longest diameter)  N1: less than 4 N+ lymph node  N2: at least 4 N+ lymph node |
| Distance to mesorectal fascia | ≤ 1mm  1-2mm: mesorectal fascia at risk / threatened  > 2mm |
| EMVI | extramural vascular invasion: -/+ |

Table S9. Standardized pathological report

| Name of specimen |  |
| --- | --- |
| Length of specimen |  |
| Location of specimen |  |
| Number of tumor |  |
| Size of tumor | Largest diameter: _________cm  Other diameter: ______cm*_____cm |
| Safety margin | proximal ______ cm  distal ______ cm  circumferential ______ cm |
| Completeness of total mesorectal excision | □ Complete: Intact mesorectum with only minor irregularities of smooth mesorectal surface. No defect is deeper than 5 mm, and there is no coning toward the distal margin of the specimen. There is a smooth CRM at slicing.  □ Nearly complete: Moderate bulk to the mesorectum, but irregularity of the mesorectal surface. Moderate coning of the specimen is allowed. At no site is the muscularis propria visible, except for the insertion of the levator muscles.  □ Incomplete: Low bulk mesorectum with defects down onto the muscularis propria and/or a very irregular CRM.  □ Cannot be determined |
| Histopathologic type of invasive carcinoma | □ Adenocarcinoma, not otherwise specified  □ Low-grade (well differentiated and moderately differentiated)  □ High-grade (poorly differentiated)  □ Mucinous adenocarcinoma  □ Signet ring cell carcinoma  □ Medullary carcinoma  □ Serrated adenocarcinoma  □ Micropapillary adenocarcinoma  □ Squamous cell (epidermoid) carcinoma (excluding upwardly spreading anal tumors)  □ Adenosquamous carcinoma  □ Small cell neuroendocrine carcinoma  □ Large cell neuroendocrine carcinoma  □ Mixed neuroendocrine-non-neuroendocrine neoplasm  □ Undifferentiated carcinoma  □ Other: (specify: ___________________) |
| Depth of invasion | □ No evidence of carcinoma (pT0)  □ Intramucosal carcinoma (pTis)  □ Tumor invades the submucosa (pT1)  □ Tumor invades the muscularis propria (pT2)  □ Tumor invades through the muscularis propria into pericolorectal tissue (pT3)  □ Tumor invades through the visceral peritoneum (pT4a)  □ Tumor directly invades or adheres to adjacent organs or structures (pT4b) |
| Resection margin | Proximal margin  □ Free from carcinoma  □ Involved by carcinoma  Distal margin  □ Free from carcinoma  □ Involved by carcinoma  Circumferential margin (rectum only)  □ Free from carcinoma  □ Involved by carcinoma |
| Regional lymph node metastasis | □ No metastasis in all ______ regional lymph nodes (pN0)  □ Metastasis to ______ out of ______ regional lymph nodes (pN _______) |
| Tumor budding | □ Not identified  □ Present  □ ≤ 4 buds (low)  □ 5–9 buds (intermediate)  □ ≥ 10 buds (high)  □ Cannot be assessed (specify: ___________________ ) |
| Lymphatic (small vessel) invasion | □ Not identified  □ Present |
| Venous invasion | □ Not identified  □ Present  □ Intramural  □ Extramural |
| Perineural invasion | □ Not identified  □ Present |
| Tumor regression grade | □ Grade 0: No viable cancer cells (complete response)  □ Grade 1: Single cells or rare small groups of cancer cells (near-complete response)  □ Grade 2: Residual cancer with evident tumor regression, but more than single cells or rare small groups of cancer cells (partial response)  □ Grade 3: Extensive residual cancer with no evident tumor regression (poor or no response) |
| DNA mismatch repair immunohistochemistry | MLH1:□ Positive (retained expression)  □ Negative (loss of expression)  MSH2:□ Positive (retained expression)  □ Negative (loss of expression)  PMS2:□ Positive (retained expression)  □ Negative (loss of expression)  MSH6:□ Positive (retained expression)  □ Negative (loss of expression)  Summary: DNA mismatch repair deficiency (was/was not) observed |
| Microsatellite instability (MSI) | Summary:  □ MSI-stable (MSS)  □ MSI-low (MSI-L)  □ MSI-high (MSI-H) |
